# A counterfactual and random intercept cross‐lagged panel analysis of the effects of reading frequency on adolescent mental health in a large longitudinal study

**DOI:** 10.1002/jcv2.70075

**Published:** 2025-11-22

**Authors:** Aja Murray, Patrick Errington, Yi Yang, Helen Wright, Daniel Mirman, Ingrid Obsuth, Tom Booth, Denis Ribeaud, Manuel Eisner

**Affiliations:** ^1^ Department of Psychology University of Edinburgh Edinburgh UK; ^2^ Department of English University of Edinburgh Edinburgh UK; ^3^ School of Health in Social Science, Clinical & Health Psychology University of Edinburgh Edinburgh UK; ^4^ Jacobs Center for Productive Youth Development University of Zurich Zürich Switzerland; ^5^ Institute of Criminology University of Cambridge Cambridge UK

**Keywords:** anxiety, counterfactual analysis, depression, mental health, psychosis, reading

## Abstract

**Background:**

Reading has been proposed as a protective factor in mental health; however, evaluating this is challenging due to a lack of trials and the possibility of confounding in observational studies.

**Methods:**

We used the complementary approaches of covariate balancing propensity score weighting and random intercepts cross‐lagged panel models in the large longitudinal Zurich Project on Social Development from Childhood to Adulthood study. Outcomes were measured from age 13 to 20 for anxiety and depression and at age 20 for psychosis‐like symptoms.

**Results:**

After accounting for potential confounding there were no significant effects of reading frequency on adolescent mental health across either approach.

**Conclusion:**

Increasing reading frequency without considering motivation, content and style of reading engagement is not a promising protective factor in adolescent mental health.

## Key Points

**What's known?**

- Reading has been proposed to be a promising scale‐able intervention to improve adolescent mental health
- It is difficult to test this claim because there are no large‐scale trials, and observational data is vulnerable to confounding

**What's new?**

- We use two complementary approaches to help overcome the issue of confounding in a large longitudinal dataset

**What's relevant?**

- We find no evidence that merely increasing reading, without considering content, motivation or style of engagement, would improve adolescent mental health

## INTRODUCTION

Reading has been proposed as a protective factor in mental health and forms the basis of a number of ‘bibliotherapeutic’ and other reading‐based interventions (Arslan et al., 2022; Carney & Robertson, 2022; Dowrick et al., 2012). This draws on several mechanistic hypotheses, for example, that reading may promote functions foundational in mental health, such as empathy, prosociality, identity and self‐esteem, as well as providing distraction and absorption, which can be important in emotion regulation (Carney & Robertson, 2022; Mak & Fancourt, 2019, 2020b). Interest in reading‐based interventions also stems from the fact that they may be highly scale‐able, associated with low stigma, and include an enjoyable activity, overcoming key barriers to receiving and adhering to mental health support (Baumel et al., 2019).

However, reading and mental health are not randomly distributed in the population (Mak & Fancourt, 2019), making associations in observational data vulnerable to confounding. Experimental studies on their links are logistically challenging and expensive, and correspondingly rare. A small number of studies have sought to address this challenge using counterfactual analysis approaches (Mak & Fancourt, 2019, 2020a, 2020b). Counterfactual analysis is grounded in the ‘potential outcomes’ framework which compares an observed outcome for an individual to their counterfactual outcome(s), that is, the outcome(s) that would have occurred had their treatment assignment been different. Since, in reality, we only observe one outcome corresponding to the treatment a person actually received, the causal effect must instead be estimated by a comparison of treated and untreated groups who are matched, re‐weighted, stratified, or otherwise balanced with respect to their confounding variables. For example, in propensity score matching, individuals similar in their ‘propensity’ for experiencing a treatment but differing in their actual experience of that treatment are compared on the outcome(s) of interest.

Using a propensity score matching approach in data from the Millennium Cohort Study, Mak and Fancourt (2019) found that reading for pleasure at age 11 was associated with higher self‐esteem. Following a similar approach in the same dataset, reading for pleasure at age 7 was associated with lower levels of attention deficit hyperactivity disorder (ADHD) symptoms and emotional problems at age 11, as well as higher levels of prosociality (Mak & Fancourt, 2020a). There was no effect on peer problems or conduct problems. Whilst this study examined emotional problems using the Strengths and Difficulties Questionnaire (SDQ; which includes items related to anxiety and depression), no studies to date have used this approach to directly examine anxiety, depression, and psychosis.

Since reading engagement may show change over development (including in response to changes in mental health, creating reciprocal effects), complementary approaches that leverage within‐person change in reading engagement and examine corresponding changes in mental health are also helpful for illuminating this association. For example, the random intercepts cross‐lagged panel model (RI‐CLPM) can provide information about how within‐person variation in a candidate treatment variable (e.g., reading) is reciprocally related to within‐person changes in an outcome variable (e.g., mental health) (Hamaker et al., 2015).

Utilising and comparing the findings from complementary approaches such as propensity‐based matching or weighting and RI‐CLPM can provide a form of triangulation of evidence and assist in the illumination of biases from each approach. Given the complexities of causal inference in human behaviour, triangulation is recommended as a means of gaining insights into the robustness and biasing influences on causal estimates (Bailey et al., 2024), for example, findings from statistical approaches such as propensity score weighting can be compared with design‐based approaches such as randomised controlled trials (Hammerton & Munafò, 2021). In the current context we compare two statistical approaches. Specifically, propensity score approaches aim to comprehensively measure and model between‐person confounds (which could be time‐stable or time‐varying). They include balance checking and/or can incorporate conditions into the estimation to help ensure balance across different levels of a treatments. They may; however, miss some unmeasured confounds and, therefore, produce biased estimates due to residual confounding. A primary advantage of the RI‐CLPM approach is that they can in principle address unmeasured confounds that are stable across time. However, they remain vulnerable to biases such as unmeasured time‐varying confounds, confounding effects due to delayed effects when only lag‐1 effects are modelled, and they require the time‐series effects to be stable over time for unbiased treatment effect estimates (Lüdtke & Robitzsch, 2022; Murayama & Gfrörer, 2022). Extensions to address some of these limitations for example, including time‐varying confounders, carry practical limitations due to the attendant increases in model complexity. There may also be a risk that RI‐CLPMs ‘over‐adjust’ for time‐stable variables given that not all stable trait‐like characteristics that could be associated with reading and mental health are necessarily confounders. Given these different ways of dealing with confounding and their associated vulnerabilities to biases, if the approaches disagree, the nature of the disagreement can be helpful for pinpointing and addressing sources of bias.

As well as methodological challenges, past research has also left it unclear whether reading remains a protective factor in and across adolescence. Adolescence is a particularly critical period for mental health, being a time of peak vulnerability for the onset and escalation of mental health issues (Kessler et al., 2005; Murray et al., 2021). Mental health issues at this stage often persist into adulthood where they can lead to lifelong distress, impairment and disadvantage (Fergusson et al., 2007; Gargano et al., 2018; Johnson et al., 2009). Conversely, establishing positive mental health‐related behaviours and habits in adolescence can help minimise lifelong risk. Given this, the goal of the current study was to establish whether reading in adolescence can support better mental health. Specifically, we sought to examine whether frequency of reading (books and magazines) during the week was causally related to subsequent mental health in adolescence. This can provide insights into whether simple cost‐effective interventions that merely seek to increase reading time during the week, without considering other features such as the content, have the potential to positively influence mental health.

## METHODS

### Participants

Participants were from the longitudinal Zurich Project on Social Development from Childhood to Adulthood (z‐proso) study (Ribeaud et al., 2022a). Z‐proso began in 2004 when participants were aged 7. Here we focused on the most recent waves of data with mental health outcomes, when participants were aged 13 (*N* = 1137), 15 (*N* = 1362), 17 (*N* = 1444) and 20 (*N* = 1301). Information on the sociodemographic composition of the sample and Ns available for each variable (smallest *N* = 762) are provided in Appendix S1. In brief, for the covariate balancing generalised propensity score (CBGPS) analyses, *n* = 1444 participants could be included in the age 17 outcome analyses and *n* = 1301 could be included in the age 20 outcome analyses based on the number of participants with complete treatment variable (reading engagement) data. In the RI‐CLPMs *n* = 1522 participants were included based on participants who contributed data for at least one time‐point in these models.

In terms of the adequacy of these samples sizes, necessary samples sizes for weighting‐based counterfactual analysis approaches depend on the degree of propensity score overlap, which can be difficult to estimate in advance (Austin, 2021). However, based on a range of plausible values, and using the approach suggested by Liu et al. (2025) we estimated that with our fixed minimum sample size of *n* = 1,301, a conservative estimate of the minimum detectable effect in terms of a standardised treatment effect would be ∼0.3 for power = 0.80 and alpha = 0.05. Previous Monte Carlo power analyses have examined the minimum effect sizes for cross‐lagged within‐person effects that can be detected with the current dataset in RI‐CLPMs (Zhu et al., 2022) with power = 0.80 and alpha = 0.05. These analyses suggested that the minimum detectable effect is equivalent to a Pearson's correlation of around *r* = 0.15 for the within‐person cross‐lagged effects.

The sample included approximately equal numbers of males and females and were from a wide range of sociodemographic backgrounds, reflecting the diversity of the population of Zurich, Switzerland. Previous research has suggested that the sample achieved good representativeness of the underlying population and has been subject to little non‐random attrition over time (Eisner et al., 2018). In terms of the current study outcomes, we examined the links between baseline parent‐reported child anxiety and depression (there were no baseline measures of psychosis) and drop‐out. Our analyses did not indicate the presence of non‐random drop‐out with respect to the outcomes of interest in the current study between baseline and the period covered by the analytic sample. Detailed information on z‐proso can be found on its website regarding recruitment, sample descriptions, and measurements (Ribeaud et al., 2022b).

For age 17 anxiety and depression we had treatment (reading engagement frequency) data at age 15 and covariate data at age 13. In addition, for age 20 anxiety, depression, and psychosis‐like symptoms, we had treatment (reading engagement) data at age 17 and covariate data at age 15. We, therefore, conducted two sets of analyses using data from the age 13–17 waves and age 15–20 waves.

For the RI‐CLPM analysis, exposure and outcome data were available for 4 waves (age 11, 13, 15, 17), allowing us to examine the anxiety and depression outcomes at the latter three waves. As psychosis‐like symptoms were only available at age 20 only the effects of reading on depression and anxiety could be explored using this approach.

### Ethics statement

In accordance with Swiss regulations, ethical approval before 2017 was not required for the minimally intrusive nature of the questions and interventions of the study. Since 2017, the main study procedures have been approved by the Ethics Committee at the Faculty of Arts and Social Sciences of the University of Zurich. Caregivers provided informed consent for the participation of their eligible children until they reached the legal age at which young people could provide consent for themselves, after which informed consent was obtained directly from the participants.

### Measures

#### Outcomes

*Anxiety and depression* were measured using the self‐reported Social Behaviour Questionnaire (SBQ; Tremblay et al., 1991) administered at the age 11–20 waves of z‐proso. It includes four items each for anxiety and depression. At age 20 it was expanded and nine items captured depression (e.g., unhappiness, lethargy). Responses were recorded on a five‐point Likert‐type scale from *never* to *very often*. Previous psychometric analyses have supported the reliability and validity of the scores from the SBQ in the current sample (Murray, Eisner, Obsuth, et al., 2017; Murray, Eisner, & Ribeaud, 2017; Murray, Obsuth, et al., 2017; Murray et al., 2019). Omega reliability for anxiety and depression at age 11 was 0.69 and 0.65 respectively; at age 13 was 0.72 and 0.74; at age 15 was 0.74 and 0.74; at age 17 was 0.77 and 0.77 and at age 20 was 0.80 and 0.90. For the RI‐CLPM analyses, only the subset of items that were consistently measured over time were used. For the CBGPS analyses, all available items were used.

*Psychosis‐like symptoms* were measured using an abbreviated six‐item version of the Community Assessment of Psychic Experiences Scale (Mark & Toulopoulou, 2016) at age 20 only. Psychotic‐like experiences were measured with six items (e.g., hearing voices). Omega reliability was 0.73.

### Treatment variable

Reading frequency was measured using a single‐item self‐report of reading frequency at the ages 11, 13, 15 and 17 waves of z‐proso and asks participants how often they ‘read a book or magazine’ on a five‐point scale from *never* to *(almost) every day* between Monday and Friday.

In the main analyses, the treatment variable was treated as continuous because evidence suggests that provided a variable has at least five (utilised) response options which approximate a normal distribution it is justifiable to treat it as continuous (Rhemtulla et al., 2012). This has the benefit of allowing us to leverage full information maximum likelihood (FIML) for missing data in the RI‐CLPM analysis and avoids discarding information.

### Counterfactual analysis matching variables

Matching variables were selected based on plausibly being a confounder, that is, having a causal impact on both the treatment and outcome variable, or on being a proxy for an unmeasured confounder (VanderWeele, 2019). The specific measures used for matching are comprehensively described in Appendix S1 and included gender, migration background, parental occupational prestige, internalising problems, ADHD symptoms, general trust, self‐control, substance use, aggression, prosociality, delinquency, parental involvement, social support, bullying victimisation, teacher bond, class bond, school difficulties, and school achievement in German, maths, and school motivation. To ensure they could not have been impacted by the treatment, all matching variables used for the age 20 outcome analyses were measured prior to age 17 (at the age 15 wave or earlier) and all covariates used for the age 17 outcomes were measured at or prior to age 13, except International socioeconomic index of occupational status occupational prestige scores and migration status, which were based on data from both age 13 and 15. School achievement variables were measured at age 12 because once youth transition to secondary school, achievement ratings are only valid when comparing youth within the same stream.

### Statistical procedure

R code is provided at the Open Science Framework (OSF).

#### Missingness

Unless data are missing completely at random, treatment effects estimated will be biased and less efficient than alternative methods that attempt to model the missingness (Leyrat et al., 2019; Rubin, 1976). We, therefore, where possible implemented methods that can provide unbiased parameter estimates under a ‘missing at random’ (MAR) assumption (see Bottigliengo et al., 2021). The MAR assumption is that missingness is random conditional on the modelled variables, that is, that missingness/drop‐out within the period studied is predictable based on other variables that enter the analysis. For our counterfactual analyses, we used multiple imputation using a ‘within’ approach that conducts the propensity score analysis within each dataset and pools the results using Rubin's rules. For this, we used a Bayesian multivariate chained equations approach (a fully conditional specification approach) implemented in the mice package in R statistical software (Buuren & Groothuis‐Oudshoorn, 2010), with 10 imputations. All matching and outcome variables were included in the imputation model (Crowe et al., 2010); however, we focused on the cases with complete treatment data, given that missingness in these variables was minimal. All variables in the imputation model were predicted from all other covariates measured at all waves using predictive mean matching. A separate imputation model was built for each set of outcome analyses (age 17 vs. 20). For the RI‐CLPM analysis, we used FIML estimation to deal with missingness, another approach which provides unbiased parameter estimates when the MAR assumption is fulfiled. This allowed a sample size of *n* = 1522 to be utilised.

#### Counterfactual analyses

We conducted linear regressions to estimate the unadjusted effects of reading engagement frequency on next‐wave anxiety, depression, and psychosis outcomes (as available). We then conducted counterfactual analyses adjusting for hypothesised pre‐treatment confounders using a generalised CBGPS approach and following the DigiCAT workflow (Murray et al., 2024). CBGPS uses a weighting‐based approach to re‐balancing cases with different levels of reading frequency, similar to an inverse probability of treatment weighting approach (Austin & Stuart, 2015). CBGPS maintains the (approximate) continuous measure scale of the treatment variable and uses a method of moments approach to propensity score estimation to minimise the association between pre‐treatment matching variables and treatment assignment. This is conceptually similar to re‐balancing a treatment and control groups with respect to a set of matching variables but represents an extension to continuous treatments. Balance can be checked by assessing the empirical weighted correlations between treatment variable and each balancing variable, with a correlation <|0.1| taken to be indicative of minimal confounding. The resulting propensity scores are used to form weights for use in weighted regression in which the treatment variable (reading) is used to predict the outcomes (subsequent anxiety, depression, and psychosis). The average treatment effect (ATE) can be obtained from this model. It is here estimated in terms of the raw units of the treatment and outcome variables and therefore represents the unit increase in anxiety, depression, or psychosis (moving from an average symptom level of e.g., ‘*often*’ to *‘very often’*) for every unit increase in reading frequency (e.g., moving from *‘never’* to *‘less than once a week’*). We used a separate weighted linear regression for each outcome: anxiety, depression, and psychosis.

As an additional (robustness) check, we also conducted variants of the analysis in which the reading variable was dichotomised (those who answered ‘never’ or ‘less than once a week’ were designated the control group and those who answered ‘on 1–2 days’, ‘on 3–4 days’, or ‘(almost) every day’ were designated the treated group). We then used propensity score matching to estimate the average effect of treatment on the treated (ATT). Details of these analyses are provided in the Appendix S2.

#### RI‐CLPM

RI‐CLPMs were fit to the longitudinal reading engagement frequency and mental health data. The RI‐CLPM can be thought of as an extension of the standard CLPM, which models the lag‐1 autoregressive and cross‐lagged relations between constructs over time. However, the standard CLPM conflates between‐person and within‐person effects, where it is the latter that is typically of interest in developmental theory and for informing interventions (Hamaker et al., 2015). The RI‐CLPM was proposed as a way to disaggregate these between‐ and within‐person effects. It does so by specifying random intercept factors for each variable (person random effects representing time‐stable individual differences in the overall levels of each variable, or ‘stable traits’) and fitting the cross‐lagged structure to the resulting time‐specific residuals. The random intercept factors are allowed to covary; they estimate the between‐person association between constructs (e.g., the extent to which people with overall higher levels of reading frequency tend to have lower overall levels of mental health issues). The time‐specific residuals after modelling these random intercepts represent individuals' deviations from their baseline level of a construct and can be used to test whether being higher or lower than usual on one construct is associated with escalations or declines in another. For example, it can be used to test whether reading more at a given time is associated with better mental health concurrently and at the next wave. This latter effect represents the within‐person cross‐lagged effects of reading on mental health. All lag‐1 autoregressive and cross‐lagged effects between the reading and relevant mental health variables were modelled, as were the concurrent (residual) covariances between reading and the relevant mental health variable at each time point. The autoregressive and cross‐lagged effects over different lags were allowed to differ (i.e., they were not constrained to equality), reflecting the possibility that reading may have different effects at different stages of development. No constraints on the mean structure were imposed (e.g., as in the autoregressive latent trajectory model with structured residuals where slopes are specified) as it has previously been highlighted that when the goal is causal inference, this risks any mis‐specification of the mean structure biasing the cross‐lagged effects (Murayama & Gfrörer, 2022).

Models were fit using the lavaan package (Rosseel, 2012) in R statistical software with maximum likelihood estimation and separate models fit for each mental health outcome. The mental health outcomes were included in the models as observed composite scores. As a robustness check, a second variant of the analysis was conducted where latent measurement models for the mental health outcomes were specified with random intercepts included for each depression/anxiety indicator. Latent within‐person factors were specified for each time‐point and longitudinal metric invariance was assumed in order to reduce the model complexity and thereby facilitate convergence following initial convergence issues.

## RESULTS

The pattern of findings in the additional (robustness) analyses was the same as for the main analysis reported below (see Appendix S2).

### Descriptive statistics

Descriptive statistics for reading frequency and the mental health outcomes are provided in Table 1. They suggest a decrease in leisure‐time reading frequency over adolescence accompanied by overall increases in anxiety and depression. Descriptive statistics for the matching variables are provided in Tables S1 and S2.

**TABLE 1:** Descriptive statistics for mental health and reading engagement variables.

Mental health outcomes					
	*N*	Mean	Min	Max	
Age 11 anxiety	1126	8.18	4	20	
Age 13 anxiety	1350	8.59	4	20	
Age 15 anxiety	1436	9.17	4	20	
Age 17 anxiety	1295	9.50	4	20	
Age 20 anxiety	1180	9.61	4	20	
Age 11 depression	1129	8.09	4	19	
Age 13 depression	1354	8.88	4	20	
Age 15 depression	1442	9.49	4	20	
Age 17 depression	1299	9.80	4	20	
Age 20 depression	1179	20.31	9	45	
Age 20 psychosis	1177	8.79	6	30	

Reading frequency					
	Never	Less than once a week	1–2 days per week	3‐4 times per week	(Almost) daily
Age 11 reading	73	180	286	206	392
Age 13 reading	257	401	325	158	221
Age 15 reading	431	491	285	118	119
Age 17 reading	427	456	252	93	73

*Note*: The possible score range for age 11–20 anxiety and age 11–17 depression is 4‐20; for age 20 depression it is 4‐45 and for age 20 psychosis it is 4–30.

### Unadjusted regressions

Coefficients for the regression of the mental health outcomes on prior‐wave reading levels without any matching variable adjustment are provided in Table 2 and suggested significant (*p* < 0.05) detrimental effects of increased leisure time reading on anxiety but no effects on depression and psychosis, except age 20 depression.

**TABLE 2:** Unadjusted regression coefficients.

Estimate	*B*	SE	*p*	*β*
Age 11 reading effects on age 13 anxiety	0.27	0.08	<0.001	0.11
Age 13 reading effects on age 15 anxiety	0.16	0.07	0.024	0.06
Age 15 reading effects on age 17 anxiety	0.24	0.08	0.004	0.08
Age 17 reading effects on age 20 anxiety	0.28	0.10	0.004	0.09
Age 11 reading effects on age 13 depression	0.10	0.08	0.18	0.04
Age 13 reading effects on age 15 depression	0.12	0.06	0.09	0.09
Age 15 reading effects on age 17 depression	0.10	0.08	0.20	0.04
Age 17 reading effects on age 20 depression	0.20	0.09	0.02	0.07
Age 17 reading effects on age 20 psychosis	−0.03	0.08	0.64	−0.01

*Note*: Each row represents the result from a separate linear regression model with leisure time reading at wave *t* − 1 as the sole predictor and mental health outcome at wave *t* as the outcome. *B* is the unstandardised regression coefficient. *β* = standardised regression coefficient.

### CBGPS

#### Age 17 outcomes analyses

All weighted matching variable and treatment correlations were close to zero (all were <0.001; see Table S3 for the full set of unweighted and CBGPS‐weighted correlations) and the average adjusted effective sample size across imputations was 815.69. Weighting substantially reduced the sample size (with attendant reductions in precision) compared with an unadjusted sample size of *n* = 1444. However, we did not trim larger weights to increase precision as retaining good balance was more important in the context of the present analyses. Using the resulting weights, there was no significant effect of age 15 reading on age 17 anxiety (*b* = 0.06, *p* = 0.50; SE = 0.089; *β* = 0.02) and nor age 17 depression (*b* = −0.08, SE = 0.085, *p* = 0.32; *β* = −0.03).

#### Age 20 outcome analyses

All weighted matching variable and treatment correlations were close to zero (see Table S4) and the average adjusted effective sample size across imputations was 605.77, compared to the unadjusted sample size of *n* = 1301. Based on the same logic as for the age 17 outcome analyses, we did not trim more extreme weights. There was no significant effect of age 17 reading on age 20 anxiety (*b* = −0.09, SE = 0.11, *p* = 0.39; *β* = −0.03), nor on depression (*b* = −0.16, SE = 0.20; *p* = 0.43; *β* = −0.03) or psychosis (*b* = −0.11, SE = 0.10; *p* = 0.23; *β* = −0.04).

#### RI‐CLPM

The RI‐CLPM for reading and anxiety fit well [Comparative Fit Index (CFI) = 0.991, Tucker‐Lewis Index (TLI) = 0.973, Root Mean Square Error of Approximation (RMSEA) = 0.040, Standardised Root Mean Residual (SRMR) = 0.021]. As the purpose of the RI‐CLPM was to provide a complementary approach to estimating the effect of reading on mental health, we focus on these coefficients here (Table 3), with full results provided at: OSF | RICLPM results.xlsx. A path diagram (Figure S1) providing key model parameters is included in Supporting Information S1. There were no significant within‐person effects of reading on anxiety, nor any significant concurrent (same‐wave) within‐person (residual) associations. This same pattern of findings was found when specifying anxiety using a latent measurement model (see: OSF).

**TABLE 3:** Coefficients for effects of reading on mental health from RI‐CLPMs.

Estimate	*B*	SE	*p*	*β*
Reading and anxiety RI‐CLPM				
Age 11 reading effects on age 13 anxiety	0.056	0.111	0.616	0.023
Age 13 reading effects on age 15 anxiety	−0.094	0.093	0.312	−0.036
Age 15 reading effects on age 17 anxiety	0.017	0.099	0.862	0.006
Reading and depression RI‐CLPM				
Age 11 reading effects on age 13 depression	−0.103	0.109	0.341	−0.043
Age 13 reading effects on age 15 depression	−0.065	0.089	0.464	−0.025
Age 15 reading effects on age 17 depression	−0.056	0.099	0.575	−0.018

*Note*: *B* is the unstandardised cross‐lagged coefficient and *β* is the standardised cross‐lagged coefficient.

The RI‐CLPM for reading and depression fit well (CFI = 0.991, TLI = 0.973, RMSEA = 0.039, SRMR = 0.019). Full results are provided at: OSF | RICLPM results.xlsx with a path diagram (Figure S2) including key parameters provided in Supporting Information S1. There were no significant within‐person effects of reading on depression, nor any significant concurrent (same‐wave) within‐person (residual) associations (Table 3). This same pattern of findings was found when specifying depression using a latent measurement model (see: OSF).

#### Comparison of approaches

Table 4 summarises the findings across the approaches, with the RI‐CLPM and CBGPS forming a set of constructive replications (note that the coefficients cannot be directly compared because they utilised different sets of items). The comparison shows that despite indications for a detrimental effect of reading on anxiety in the unadjusted analyses, the two complementary adjusted analyses (CBGPS and RI‐CLPM) were in agreement in suggesting no significant effects of reading on mental health.

**TABLE 4:** Summary of findings on the effect of prior reading engagement on mental health outcomes across approaches.

	Unadjusted linear regression	CBGPS	RI‐CLPM
Age 13 anxiety	*β* = 0.11 (*p* < 0.001)*	Not tested	*β* = 0.02 (*p* = 0.62)
Age 15 anxiety	*β* = 0.06 (*p* = 0.024)*	Not tested	*β* = −0.04 (*p* = 0.31)
Age 17 anxiety	*β* = 0.08 (*p* = 0.004)*	*β* = 0.02 (*p* = 0.50)	*β* = 0.01 (*p* = 0.86)
Age 20 anxiety	*β* = 0.09 (*p* = 0.004)*	*β* = −0.03 (*p* = 0.32)	Not tested
Age 13 depression	*β* = 0.04 (*p* = 0.18)	Not tested	*β* = −0.04 *(p* = 0.34)
Age 15 depression	*β* = 0.09 (*p* = 0.09)	Not tested	*β* = −0.03 (*p* = 0.46)
Age 17 depression	*β* = 0.04 (*p* = 0.20)	*β* = −0.03 (*p* = 0.39)	*β* = −0.02 (*p* = 0.58)
Age 20 depression	*β* = 0.07 (*p* = 0.02)*	*β* = −0.03 (*p* = 0.43)	Not tested
Age 20 psychosis	*β* = −0.01 (*p* = 0.64)	*β* = −0.04 (*p* = 0.23)	Not tested

*Note*: *β* is the standardised regression coefficient.

*Significant at *p* < 0.05.

## DISCUSSION

In this study we examined whether reading frequency in adolescence is a protective factor in mental health. We found no evidence for an effect of frequency of weekday leisure time reading on adolescent anxiety, depression or psychosis once adjusting for confounding using CBGPS and RI‐CLPM approaches. Taken together, results suggest that for this age range, general leisure time reading during the week—without consideration of the content or nature of engagement—is not a promising target for intervention to improve adolescent mental health.

Given the high prevalence of adolescent mental health issues and associated pressures on mental health services, there is a strong need to identify potential preventive interventions that can be delivered at scale. This has stimulated interest in reading as a potential intervention. As noted; however, analysing the impact of reading for pleasure on mental health must take into account confounding due to the social patterning of reading for pleasure (Mak & Fancourt, 2019). For example, factors such as language ability, attention difficulties, socioeconomic background, and pre‐existing mental health symptoms may impact motivation and/or ability to engage with reading and subsequent mental health. Counterfactual methods complemented by RI‐CLPMs are valuable for providing a solution to this issue when only observational data are available and can help inform which protective factors are worth further exploring in interventions.

Previous counterfactual analysis studies have identified an effect of reading for pleasure on mental health up to early adolescence (Mak & Fancourt, 2019, 2020a). However, our own study which analysed effects across adolescence suggested no beneficial effect. There are various potential explanations for these findings, including the possibility that the previous study captured reading effects at a critical period of development and the effects of reading are less important later in adolescence. However, there were also a range of methodological differences: the MCS study is larger (they analysed data from *n* = 8936 participants), therefore, power is likely greater. However, the previous studies also adjusted for a less comprehensive confounder set, therefore, could be more vulnerable to confounding bias, especially because prior levels of child mental health issues were not adjusted for. The treatment and outcome variables were also measured differently (mental health was measured by the SDQ; Goodman, 1997) and by different informants (parent reports were used in the previous study) which may have contributed to the discrepancy. Finally, the estimand differed as we focused on the ATE, whereas the previous authors focused on the ATT. It is also possible that the setting (Switzerland vs. the UK) played a role in the differences.

In contrast to the findings of the previous study, our findings are consistent with the notion that reading—without regard for motivation, content, and style of engagement—is not in itself a promising intervention for mental health symptoms. Indeed, other studies have begun to uncover the roles of factors such as autonomous motivation (Levine et al., 2022), content (Arslan et al., 2022), and form of engagement (Carney & Robertson, 2022). Building on these and our own findings, future studies could gather more detailed information about naturalistic reading behaviours to illuminate whether there are potential ‘active ingredients’ in reading that could form the basis for scale‐able interventions for mental health. For example, preliminary evidence suggested that reading positive psychology‐based stories may have a positive impact on mental health in young readers (Arslan et al., 2022). Research could likewise examine potential detrimental aspects of reading, for example, exposure to content which is unhelpful for mental health, such as content which promotes negative self‐evaluations (e.g., by presenting unrealistic standards) or unhelpful ways of thinking and behaving.

### Limitations

It is important to note the limitations of the current study. First, due to the time lags, we could only detect effects persisting over a 2‐ or 3‐year period and future research could help clarify whether leisure time reading has a short‐term impact on mental health.

Second, counterfactual methods rely on several assumptions which are typically untestable. First, there is an assumption of ‘no interference’ (i.e., treatment assignment for units does not affect the potential outcomes of others) and that there is only a single version of the treatment. Together these form the ‘stable unit treatment value assignment’ assumption. Second, independence of the potential outcomes of the treatment assignment conditional on the modelled confounders is assumed (i.e., unconfoundedness) and positivity (each unit has a positive probability of receiving a treatment at all levels of the confounders) are assumed, together forming the ‘strong ignorability’ assumption. Consistency (the potential outcome under a given treatment is equal to the observed outcome if the actual treatment is received) is also assumed.

While these assumptions cannot usually be directly formally tested, it is possible to consider their plausibility. For example, assessing covariate balance combined with background substantive knowledge of whether it was possible to capture all relevant confounders can inform the assessment of the unconfoundedness assumption. In the present analysis arguably the main threat in terms of assumption violations is the possibility that relevant confounding effects were not directly included in our models (and were only incompletely captured via their correlations with included variables). For example, socioeconomic status was represented only by a parental occupational prestige variable due to data availability and variables such as physical activity, intelligence and screen time could be additional potential confounders which we did not include. In terms of the complementary RI‐CLPM we used, this is vulnerable to the effects of different biases, such as within‐person time‐varying confounds.

Our measure of reading was coarse, and we were unable to examine the effects of content, style of engagement, motivation, or other aspects of how adolescents engage with reading on mental health. Thus, our treatment effect was not precisely defined and thus only provides potential insights into how very simple interventions that merely attempt to increase reading frequency during the week might perform in reducing later mental health issues. As we only had a measure of psychosis‐like symptoms at age 20, we could not examine the impact of reading on this outcome at earlier ages. Finally, power analyses suggest that both our RI‐CLPM analyses and weighting‐based analyses were not powered to detect effect sizes less than those equivalent to a Pearson correlation of around *r* = 0.15 (the standardised effect of 0.30 for the latter converts to ∼ *r* = 0.15). Whilst effect sizes smaller than these could be arguably to be too small to be clinically significant, it may be that even small effect sizes produced by a simple intervention that can be scaled to a population level could be considered meaningful.

### Future directions

The current study employed two complementary approaches drawn from propensity weighting and structural equation model (SEM) frameworks to assess the effects of reading frequency on mental health in adolescence. However, overall, there has been little explicit comparison and/or integration of these approaches. Further development of this literature would be valuable for supporting the use of evidence triangulation in establishing the effects of candidate treatments on mental health outcomes over development. It would also serve to strengthen understanding of the biases that arise in real‐world applications of the approaches. This could build on previous illuminating work that has discussed SEM‐based approaches in terms of the potential outcomes framework and/or their ability to recover causal effects under different specifications and assumptions (Lüdtke & Robitzsch, 2022; Murayama & Gfrörer, 2022). Of particular interest would be the further comparison of the widely used RI‐CLPM and its various extensions with other SEM‐based approaches that aim to or have been claimed to account for unobserved confounding, such as the dynamic panel model and autoregressive latent trajectory models.

### Conclusions

Weekday leisure time reading in adolescence was not related to later mental health. This suggests promoting general reading for pleasure without consideration of content or style of engagement may not be a promising intervention target for long‐term effects on mental health in this age group.

## AUTHOR CONTRIBUTIONS

**Aja Murray**: Conceptualization; data curation; formal analysis; investigation; methodology; resources; software; supervision; validation; visualization; writing—original draft; writing—review and editing. **Patrick Errington**: Conceptualization; writing—original draft; writing—review and editing. **Yi Yang**: Methodology; writing—original draft; writing—review and editing. **Helen Wright**: Methodology; writing—original draft; writing—review and editing. **Daniel Mirman**: Validation; writing—original draft; writing—review and editing. **Ingrid Obsuth**: Validation; writing—original draft; writing—review and editing. **Tom Booth**: Validation; writing—original draft; writing—review and editing. **Denis Ribeaud**: Funding acquisition; project administration; validation; writing—review and editing. **Manuel Eisner**: Funding acquisition; project administration; validation; writing—review and editing.

## CONFLICT OF INTEREST STATEMENT

The authors declare no conflicts of interest.

## ETHICAL CONSIDERATIONS

In accordance with Swiss regulations, ethical approval before 2017 was not required for the minimally intrusive nature of the questions and interventions of the study. Since 2017, the main study procedures have been approved by the Ethics Committee at the Faculty of Arts and Social Sciences of the University of Zurich (approval number: 21.12.13; approval date: 20/01/2022). Caregivers provided informed consent for the participation of their eligible children until they reached the legal age at which young people could provide consent for themselves. After this, informed consent was obtained directly from the participants.

## Supporting information

Supporting Information S1

## Data Availability

The data that support the findings of this study are available from author Denis Ribeaud upon reasonable request.

## References

[jcv270075-bib-0001] Arslan, G. , Yıldırım, M. , Zangeneh, M. , & Ak, İ. (2022). Benefits of positive psychology‐based story reading on adolescent mental health and well‐being. Child Indicators Research, 15(3), 1–13. 10.1007/s12187-021-09891-4 PMC8731136 35013685

[jcv270075-bib-0002] Austin, P. C. (2021). Informing power and sample size calculations when using inverse probability of treatment weighting using the propensity score. Statistics in Medicine, 40(27), 6150–6163. 10.1002/sim.9176 34510501 PMC9293235

[jcv270075-bib-0003] Austin, P. C. , & Stuart, E. A. (2015). Moving towards best practice when using inverse probability of treatment weighting (IPTW) using the propensity score to estimate causal treatment effects in observational studies. Statistics in Medicine, 34(28), 3661–3679. 10.1002/sim.6607 26238958 PMC4626409

[jcv270075-bib-0004] Bailey, D. H. , Jung, A. J. , Beltz, A. M. , Eronen, M. I. , Gische, C. , Hamaker, E. L. , Kording, K. P. , Lebel, C. , Lindquist, M. A. , Moeller, J. , Razi, A. , Rohrer, J. M. , Zhang, B. , & Murayama, K. (2024). Causal inference on human behaviour. Nature Human Behaviour, 8(8), 1448–1459. 10.1038/s41562-024-01939-z 39179747

[jcv270075-bib-0005] Baumel, A. , Muench, F. , Edan, S. , & Kane, J. M. (2019). Objective user engagement with mental health apps: Systematic search and panel‐based usage analysis. Journal of Medical Internet Research, 21(9), e14567. 10.2196/14567 31573916 PMC6785720

[jcv270075-bib-0006] Bottigliengo, D. , Lorenzoni, G. , Ocagli, H. , Martinato, M. , Berchialla, P. , & Gregori, D. (2021). Propensity score analysis with partially observed baseline covariates: A practical comparison of methods for handling missing data. International Journal of Environmental Research and Public Health, 18(13), 6694. 10.3390/ijerph18136694 34206234 PMC8293809

[jcv270075-bib-0007] Buuren, S. V. , & Groothuis‐Oudshoorn, K. (2010). mice: Multivariate imputation by chained equations in R. Journal of Statistical Software, 45(3), 1–68. 10.18637/jss.v045.i03

[jcv270075-bib-0008] Carney, J. , & Robertson, C. (2022). Five studies evaluating the impact on mental health and mood of recalling, reading, and discussing fiction. PLoS One, 17(4), e0266323. 10.1371/journal.pone.0266323 35395034 PMC8993009

[jcv270075-bib-0009] Crowe, B. J. , Lipkovich, I. A. , & Wang, O. (2010). Comparison of several imputation methods for missing baseline data in propensity scores analysis of binary outcome. Pharmaceutical Statistics, 9(4), 269–279. 10.1002/pst.389 19718652

[jcv270075-bib-0010] Dowrick, C. , Billington, J. , Robinson, J. , Hamer, A. , & Williams, C. (2012). Get into reading as an intervention for common mental health problems: Exploring catalysts for change. Medical Humanities, 38(1), 15–20. 10.1136/medhum-2011-010083 22345586

[jcv270075-bib-0011] Eisner, N. L. , Murray, A. L. , Eisner, M. , & Ribeaud, D. (2018). A practical guide to the analysis of non‐response and attrition in longitudinal research using a real data example. International Journal of Behavioral Development, 0165025418797004.

[jcv270075-bib-0012] Fergusson, D. M. , Boden, J. M. , & Horwood, L. J. (2007). Recurrence of major depression in adolescence and early adulthood, and later mental health, educational and economic outcomes. The British Journal of Psychiatry, 191(4), 335–342. 10.1192/bjp.bp.107.036079 17906244

[jcv270075-bib-0013] Gargano, L. M. , Locke, S. , Li, J. , & Farfel, M. R. (2018). Behavior problems in adolescence and subsequent mental health in early adulthood: Results from the World Trade Center Health Registry Cohort. Pediatric Research, 84(2), 205–209. 10.1038/s41390-018-0050-8 29907850 PMC6185774

[jcv270075-bib-0014] Goodman, R. (1997). The Strengths and Difficulties Questionnaire: A research note. Journal of Child Psychology and Psychiatry, 38(5), 581–586. 10.1111/j.1469-7610.1997.tb01545.x 9255702

[jcv270075-bib-0015] Hamaker, E. L. , Kuiper, R. M. , & Grasman, R. P. (2015). A critique of the cross‐lagged panel model. Psychological Methods, 20(1), 102–116. 10.1037/a0038889 25822208

[jcv270075-bib-0016] Hammerton, G. , & Munafò, M. R. (2021). Causal inference with observational data: The need for triangulation of evidence. Psychological Medicine, 51(4), 563–578. 10.1017/s0033291720005127 33682654 PMC8020490

[jcv270075-bib-0017] Johnson, J. G. , Cohen, P. , & Kasen, S. (2009). Minor depression during adolescence and mental health outcomes during adulthood. The British Journal of Psychiatry, 195(3), 264–265. 10.1192/bjp.bp.108.054239 19721119 PMC2801821

[jcv270075-bib-0018] Kessler, R. C. , Berglund, P. , Demler, O. , Jin, R. , Merikangas, K. R. , & Walters, E. E. (2005). Lifetime prevalence and age‐of‐onset distributions of DSM‐IV disorders in the National Comorbidity Survey Replication. Archives of General Psychiatry, 62(6), 593–602. 10.1001/archpsyc.62.6.593 15939837

[jcv270075-bib-0019] Levine, S. L. , Cherrier, S. , Holding, A. C. , & Koestner, R. (2022). For the love of reading: Recreational reading reduces psychological distress in college students and autonomous motivation is the key. Journal of American College Health, 70(1), 158–164. 10.1080/07448481.2020.1728280 32150516

[jcv270075-bib-0020] Leyrat, C. , Seaman, S. R. , White, I. R. , Douglas, I. , Smeeth, L. , Kim, J. , Resche‐Rigon, M. , Carpenter, J. R. , & Williamson, E. J. (2019). Propensity score analysis with partially observed covariates: How should multiple imputation be used? Statistical Methods in Medical Research, 28(1), 3–19. 10.1177/0962280217713032 28573919 PMC6313366

[jcv270075-bib-0021] Liu, B. , Zhou, X. , & Li, F. (2025). Sample size and power calculation for propensity score analysis of observational studies. arXiv preprint arXiv:2501.11181. 10.48550/arXiv.2501.11181

[jcv270075-bib-0022] Lüdtke, O. , & Robitzsch, A. (2022). A comparison of different approaches for estimating cross‐lagged effects from a causal inference perspective. Structural Equation Modeling: A Multidisciplinary Journal, 29(6), 888–907. 10.1080/10705511.2022.2065278

[jcv270075-bib-0023] Mak, H. W. , & Fancourt, D. (2019). Arts engagement and self‐esteem in children: Results from a propensity score matching analysis. Annals of the New York Academy of Sciences, 1449(1), 36–45. 10.1111/nyas.14056 30985011 PMC6767447

[jcv270075-bib-0024] Mak, H. W. , & Fancourt, D. (2020a). Longitudinal associations between reading for pleasure and child maladjustment: Results from a propensity score matching analysis. Social Science & Medicine, 253, 112971. 10.1016/j.socscimed.2020.112971 32276184 PMC7429985

[jcv270075-bib-0025] Mak, H. W. , & Fancourt, D. (2020b). Reading for pleasure in childhood and adolescent healthy behaviours: Longitudinal associations using the Millennium Cohort Study. Preventive Medicine, 130, 105889. 10.1016/j.ypmed.2019.105889 31765711 PMC6983940

[jcv270075-bib-0026] Mark, W. , & Toulopoulou, T. (2016). Psychometric properties of “community assessment of psychic experiences”: Review and meta‐analyses. Schizophrenia Bulletin, 42(1), 34–44. 10.1093/schbul/sbv088 26150674 PMC4681550

[jcv270075-bib-0027] Murayama, K. , & Gfrörer, T. (2022). Thinking clearly about time‐invariant confounders in cross‐lagged panel models: A guide for model choice from causal inference perspective. https://osf.io/bt9xr/download 10.1037/met0000647 39298188

[jcv270075-bib-0028] Murray, A. L. , Booth, T. , Eisner, M. , Ribeaud, D. , McKenzie, K. , & Murray, G. (2019). An analysis of response shifts in teacher reports associated with the use of a universal school‐based intervention to reduce externalising behaviour. Prevention Science, 20, 1265–1273. 10.1007/s11121-019-00999-2 30847752 PMC6881257

[jcv270075-bib-0029] Murray, A. L. , Eisner, M. , Obsuth, I. , & Ribeaud, D. (2017). Situating violent ideations within the landscape of mental health: Associations between violent ideations and dimensions of mental health. Psychiatry Research, 249, 70–77. 10.1016/j.psychres.2017.01.005 28073033

[jcv270075-bib-0030] Murray, A. L. , Eisner, M. , & Ribeaud, D. (2017). Can the Social Behavior Questionnaire help meet the need for dimensional, transdiagnostic measures of childhood and adolescent psychopathology? European Journal of Psychological Assessment, 35(5), 674–679. 10.1027/1015-5759/a000442

[jcv270075-bib-0031] Murray, A. L. , Obsuth, I. , Eisner, M. , & Ribeaud, D. (2017). Evaluating longitudinal invariance in dimensions of mental health across adolescence: An analysis of the Social Behavior Questionnaire. Assessment, 1073191117721741. 10.1177/1073191117721741 28758417

[jcv270075-bib-0032] Murray, A. L. , Ushakova, A. , Speyer, L. , Brown, R. , Auyeung, B. , & Zhu, X. (2021). Sex/gender differences in individual and joint trajectories of common mental health symptoms in early to middle adolescence. JCPP Advances, 2(1), e12057. 10.1002/jcv2.12057 37431498 PMC10242831

[jcv270075-bib-0033] Murray, A. L. , Wright, H. , Casey, H. , Yang, Y. , Zhu, X. , Obsuth, I. , Allitt, M. , Mirman, D. , Errington, P. , & King, J. (2024). Introducing DigiCAT: A digital tool to promote the principled use of counterfactual analysis for identifying potential active ingredients in mental health. Wellcome Open Research, 9(376), 376. 10.12688/wellcomeopenres.21105.1

[jcv270075-bib-0034] Rhemtulla, M. , Brosseau‐Liard, P. É. , & Savalei, V. (2012). When can categorical variables be treated as continuous? A comparison of robust continuous and categorical SEM estimation methods under suboptimal conditions. Psychological Methods, 17(3), 354–373. 10.1037/a0029315 22799625

[jcv270075-bib-0035] Ribeaud, D. , Murray, A. , Shanahan, L. , Shanahan, M. J. , & Eisner, M. (2022a). Cohort profile: The Zurich Project on the Social Development from Childhood to Adulthood (z‐proso). Journal of Developmental and Life‐Course Criminology, 8(1), 151–171. 10.1007/s40865-022-00195-x 35223378 PMC8860297

[jcv270075-bib-0036] Ribeaud, D. , Murray, A. , Shanahan, L. , Shanahan, M. J. , & Eisner, M. (2022b). Cohort profile: The Zurich Project on the Social Development from Childhood to Adulthood (z‐proso). Journal of Developmental and Life‐Course Criminology, 8(1), 151–171. 10.1007/s40865-022-00195-x 35223378 PMC8860297

[jcv270075-bib-0037] Rosseel, Y. (2012). lavaan: An R package for structural equation modeling and more. Version 0.5–12 (BETA). Journal of Statistical Software, 48(2), 1–36.

[jcv270075-bib-0038] Rubin, D. B. (1976). Inference and missing data. Biometrika, 63(3), 581–592. 10.2307/2335739

[jcv270075-bib-0039] Tremblay, R. E. , Loeber, R. , Gagnon, C. , Charlebois, P. , Larivee, S. , & LeBlanc, M. (1991). Disruptive boys with stable and unstable high fighting behavior patterns during junior elementary school. Journal of Abnormal Child Psychology, 19(3), 285–300. 10.1007/bf00911232 1865046

[jcv270075-bib-0040] VanderWeele, T. J. (2019). Principles of confounder selection. European Journal of Epidemiology, 34(3), 211–219. 10.1007/s10654-019-00494-6 30840181 PMC6447501

[jcv270075-bib-0041] Zhu, X. , Griffiths, H. , Eisner, M. , Hepp, U. , Ribeaud, D. , & Murray, A. L. (2022). Developmental associations between bullying victimization and suicidal ideation and direct self‐injurious behavior in adolescence and emerging adulthood. Journal of Child Psychology and Psychiatry, 63(7), 820–828. 10.1111/jcpp.13529 34595760

