# Supplementary material for: A counterfactual and random intercept cross‐lagged panel analysis of the effects of reading frequency on adolescent mental health in a large longitudinal study
Source: JCPP Adv. 2025 Nov 22;6(3):e70075. doi: 10.1002/jcv2.70075 (PMC13338998; doi:10.1002/jcv2.70075)
Supplement: Supplementary file 1 [file JCV2-6-e70075-s001.docx]

**A counterfactual and random intercept cross lagged panel (RI-CLPM) analysis of the effects of reading frequency on adolescent mental health in a large longitudinal study**

**Supporting Information**

**Appendix S1. Matching Variable Measures**

*Gender* was measured based using a single binary item (‘male’ versus ‘female’).

*Migration background* was measured using a single item at waves 5 and 6 (ages 13 and 15) and coded 1= at least one parent born in Switzerland and 2= both parents born abroad.

*Socioeconomic status* was estimated based status based on the ISEI measure of *parental occupational prestige* measured at waves 5 and 6 (ages 13 and 15) (Ganzeboom et al., 1992).

*Internalising problems* were measured at ages 15 and 17 using a composite of 8 self-reported items (example: ‘*I was worried’*) from the Social Behavior Questionnaire (Murray, Obsuth, et al., 2017; Tremblay et al., 1991). These items covered the domains of anxiety and depression. Responses for all items within the SBQ were recorded on a 5-point Likert-type scale from *never* to *very often.*

*ADHD symptoms* were measured at ages 15 and 17 using a composite of 4 self-reported items from (example: ’you were restless and struggled to sit still’) from the Social Behavior Questionnaire (Tremblay et al., 1991). These items covered inattention, hyperactivity, and impulsivity.

*General trust* was measured at ages 15 and 17 using a 3-item (e.g., “most people can be trusted”) measure adapted from the *World Values Survey* (N. D. Johnson & Mislin, 2012).

*Self-control* was measured at ages 15 and 17 by 10 items (e.g., “I often act on the spur of the moment without stopping to think”) which was an adapted version of Grasmick’s Low Self-control scale (Grasmick et al., 1993; Longshore et al., 1996), measuring impulsivity, risk-seeking, preference for physical over cognitive activity, temper, and self-centredness.

*Substance use* was measured at ages 15 and 17 using 4 items measuring the lifetime prevalence (used versus not used the substance) of: tobacco use, alcohol use (beer/wine/alcopops), alcohol use (spirits), and cannabis use.

*Aggression* was measured at ages 15 and 17 in 4 sub-dimensions, each measured with 4 items: reactive, proactive, indirect, and physical aggression (example item ‘You got very angry when someone teased or irritated you’) from the self-reported Social Behavior Questionnaire (Tremblay et al., 1991). Composite scores were formed for each sub-dimension.

*Prosociality* was measured at ages 15 and 17 using 10 items (example item: ‘you tried to help someone who was hurt’) from the self-reported Social Behavior Questionnaire (Tremblay et al., 1991). These items covered empathy and helping behaviour. Composite scores were formed to provide an overall measure of prosociality.

*Delinquency* was measured at ages 15 and 17 using a variety score of 19 delinquent behaviours: truancy, cheating at school, stealing at school, stealing at home, running away from home, shoplifting goods worth less than 50 Swiss Francs, shoplifting goods worth more than 50CHF, vehicle theft, driving without a license, illegal download or upload, car/house burglary, drug dealing, fare dodging, graffiti/tags, vandalism, weapon carrying, threat/extortion, robbery, and assault. Variety scores were formed by the sum of dichotomously scored items measuring the incidence of each of these behaviours in the previous 12 months.

*Parental involvement* was measured using a composite of 7 items (example item: ‘Your parents are interested in what you do’) from the parental involvement sub-scale of the self-report version of the *Alabama Parenting Questionnaire* (APQ; Shelton et al., 1996). Responses were recorded on a 4-point Likert-type scale from *never* to *often/always.*

*Social support* was measured from adults (4 items; example item: ‘I discuss my problems with adults’) and peers (3 items: example item: ‘I get along well with my friends’) with composite scores formed for each source of social support. Responses to individual items were recorded on a 4-point Likert-type scale from *fully* *false* to *fully* *true.*

*Bullying victimisation* was measured using the 4-item *Zurich Brief Bullying Scales (ZBBS)* victimisation subscale (Murray, Eisner, et al., 2021). This measures exposure to several forms of bullying: physical aggression, property destruction, verbal aggression and social exclusion (example item: ‘…how many times have other youths laughed at you, mocked you, or insulted you?’). Responses are recorded on a 6-point Likert-type scale from *never* to *(almost) every day*. A composite score was formed to measure overall bullying victimisation exposure.

*Teacher bond* was measured using 3 items (example item: ‘I have a good relationship with my teacher’), with responses recorded on a 4-point Likert-type scale from *false* to *true.* Respondents were instructed to give an average assessment when they had more than one teacher. A composite score was formed from the 3 items to provide an overall measure of the quality of the young person’s bond to their teachers.

*Class bond* was measured using three items (example item: ‘classmates are nice to me’) with responses recorded on a 4-point Likert-type scale from *false* to *true.* A composite score was formed from the 3 items to provide an overall measure of the quality of the young person’s bond to their school class.

*School difficulties* were measured using 4 items (example item: ‘I often have bad grades’) with responses recorded on a 4-point Likert-type scale from *false* to *true.* A composite score was formed from the 4 items to provide an overall measure of the level of difficulties at school.

*School achievement* was measured based on teacher assessments in the domains of: German, maths, and school motivation.

**Appendix S2. Additional analyses**

We also examined group differences using a dichotomised reading variable using propensity score matching. We used nearest neighbour matching to match treated and control cases, based on a logistic regression propensity model. Given that we had relatively even numbers of participants in each group, we used 1:1 matching, implemented using the MatchIt package in R statistical software (Stuart et al., 2011). If successful matching could be achieved (defined by standardised mean differences between the matched treated and control groups of <|.15| for all covariates), we then used a separate linear regression for each outcome: anxiety, depression, and psychosis.

For the age 17 outcome analyses, propensity score matching with nearest neighbour matching and a logistic regression propensity model resulted in an average of 522 matched treated and control units across the imputed datasets. Most covariates had standardised mean differences (SMD) of <|.15| across the treated vs control groups, the exceptions being: ISEI (SMD= .19), parental involvement (SMD=.17), and school achievement in the domain of motivation (SMD=.16). Using these matches there was no significant effect of reading engagement on age 17 anxiety after matching and adjustment for the matching variables (b=.06, *p*=.72). There was also no effect on age 17 depression after matching and with adjustment for the matching variables (b=13, *p*=.49).

For the age 20 outcome analyses, there was an average of 418 cases successfully matched across the imputed datasets. The average standardised mean differences in covariates across group was <|.15| for all covariates, except low self-control (SMD=-0.163). Using these matches there was no significant effect of reading engagement on age 20 anxiety after adjusting for matching variables (b=.07, *p*=.75) nor on depression (b=.19, *p* = .68) nor psychosis-like symptoms (b=.31, *p*=.14).

**Table S1: Descriptive statistics for matching variables in age 17 outcome analyses**

|  | **n** | **mean** | **SD** | **min** | **max** |
| --- | --- | --- | --- | --- | --- |
| Gender | Male = 870; female = 805 | | | | |
| Migration status | At least one parent born in Switzerland = 729;  Both parents born abroad = 716 | | | | |
| ISEI (Occupational prestige) | 1332 | 45.97 | 19.16 | 16 | 90 |
| Age 13 internalising problems | 1364 | 2.19 | .73 | 1 | 5 |
| Age 13 ADHD symptoms | 1364 | 2.63 | .76 | 1 | 5 |
| Age 13 general trust | 1360 | 2.61 | .58 | 1 | 4 |
| Age 13 self-control | 1357 | 2.20 | .48 | 1 | 4 |
| Age 13 alcohol use (liquor) | 1346 | .21 | .40 | 0 | 1 |
| Age 13 alcohol use (beer/wine) | 1354 | .29 | .45 | 0 | 1 |
| Age 13 tobacco use | 1359 | .29 | .45 | 0 | 1 |
| Age 13 cannabis use | 1352 | .09 | .29 | 0 | 1 |
| Age 13 reactive aggression | 1364 | 2.12 | .69 | 1 | 5 |
| Age 13 physical aggression | 1364 | 1.63 | .79 | 1 | 5 |
| Age 13 proactive aggression | 1364 | 1.50 | .62 | 1 | 5 |
| Age 13 indirect aggression | 1364 | 1.91 | .74 | 1 | 5 |
| Age 13 prosociality | 1364 | 3.56 | .68 | 1 | 5 |
| Age 13 delinquency | 1359 | 2.90 | 2.58 | 0 | 19 |
| Age 13 parental involvement | 1361 | 3.08 | .59 | 1 | 4 |
| Age 13 social support from adults | 1360 | 2.90 | .70 | 1 | 4 |
| Age 13 social support from friends | 1360 | 3.73 | .42 | 1 | 4 |
| Age 13 bullying victimisation | 1363 | 1.70 | .77 | 1 | 5.75 |
| Age 13 teacher bond | 1360 | 3.15 | .65 | 1 | 4 |
| Age 13 class bond | 1361 | 3.29 | .61 | 1 | 4 |
| Age 13 school difficulties | 1360 | 2.00 | .55 | 1 | 4 |
| School achievement: maths | 975 | 3.25 | 1.32 | 1.00 | 5.00 |
| School achievement: German | 975 | 3.34 | 1.29 | 1.00 | 5.00 |
| School achievement: motivation | 971 | 3.51 | 1.19 | 1.00 | 5.00 |

**Table S2: Descriptive statistics for matching variables in age 20 outcome analyses**

|  | **n** | **mean** | | **SD** | **min** | **max** |
| --- | --- | --- | --- | --- | --- | --- |
| Gender | Male = 870; female = 805 | | | | | |
| Migration status | At least one parent born in Switzerland = 729;  Both parents born abroad = 716 | | | | | |
| ISEI (Occupational prestige) | 1332 | 45.97 | 19.16 | | 16 | 90 |
| Age 15 internalising problems | 1445 | 2.33 | | .78 | 1.00 | 5.00 |
| Age 15 ADHD symptoms | 1445 | 2.70 | | .78 | 1.00 | 5.00 |
| Age 15 general trust | 1444 | 2.41 | | .58 | 1.00 | 4.00 |
| Age 15 self-control | 1443 | 2.27 | | .43 | 1.00 | 3.90 |
| Age 15 alcohol use (liquor) | 1436 | .50 | | .50 | .00 | 1.00 |
| Age 15 alcohol use (beer/wine) | 1437 | .57 | | .50 | .00 | 1.00 |
| Age 15 tobacco use | 1441 | .63 | | .48 | .00 | 1.00 |
| Age 15 cannabis use | 1439 | .35 | | .48 | .00 | 1.00 |
| Age 15 reactive aggression | 1445 | 2.04 | | .67 | 1.00 | 5.00 |
| Age 15 physical aggression | 1445 | 1.52 | | .75 | 1.00 | 5.00 |
| Age 15 proactive aggression | 1445 | 1.51 | | .61 | 1.00 | 5.00 |
| Age 15 indirect aggression | 1445 | 1.76 | | .71 | 1.00 | 5.00 |
| Age 15 prosociality | 1445 | 3.60 | | .63 | 1.25 | 5.00 |
| Age 15 delinquency | 1445 | 3.59 | | 2.53 | .00 | 18.00 |
| Age 15 parental involvement | 1446 | 3.01 | | .62 | 1.00 | 4.00 |
| Age 15 social support from adults | 1560 | 2.90 | | .70 | 1.00 | 4.00 |
| Age 15 social support from friends | 1560 | 3.73 | | .42 | 1.00 | 4.00 |
| Age 15 bullying victimisation | 1444 | 1.64 | | .69 | 1.00 | 5.75 |
| Age 15 teacher bond | 1445 | 3.06 | | .67 | 1.00 | 4.00 |
| Age 15 class bond | 1445 | 3.29 | | .60 | 1.00 | 4.00 |
| Age 15 school difficulties | 1444 | 1.97 | | .55 | 1.00 | 4.00 |
| School achievement: maths | 975 | 3.25 | | 1.32 | 1.00 | 5.00 |
| School achievement: German | 975 | 3.34 | | 1.29 | 1.00 | 5.00 |
| School achievement: motivation | 971 | 3.51 | | 1.19 | 1.00 | 5.00 |

**Table S3: Correlations between matching variables and reading frequency before and after CBGPS weighting for the age 17 outcome analyses**

| **Matching variable** | **Unweighted correlation** | **Weighted correlation** |
| --- | --- | --- |
| Gender | .20 | <.001 |
| Migration status | -.15 | <.001 |
| ISEI (Occupational prestige) | .24 | <.001 |
| Age 15 internalising problems | .09 | <.001 |
| Age 15 ADHD symptoms | .03 | <.001 |
| Age 15 general trust | .04 | <.001 |
| Age 15 self-control | -.19 | <.001 |
| Age 15 alcohol use (liquor) | -.18 | <.001 |
| Age 15 alcohol use (beer/wine) | -.04 | <.001 |
| Age 15 tobacco use | -.17 | <.001 |
| Age 15 cannabis use | -.10 | <.001 |
| Age 15 reactive aggression | -.20 | <.001 |
| Age 15 physical aggression | -.23 | <.001 |
| Age 15 proactive aggression | -.17 | <.001 |
| Age 15 indirect aggression | -.18 | <.001 |
| Age 15 prosociality | .22 | <.001 |
| Age 15 delinquency | -.15 | <.001 |
| Age 15 parental involvement | .25 | <.001 |
| Age 15 social support from adults | .19 | <.001 |
| Age 15 social support from friends | .01 | <.001 |
| Age 15 bullying victimisation | .04 | <.001 |
| Age 15 teacher bond | .12 | <.001 |
| Age 15 class bond | .05 | <.001 |
| Age 15 school difficulties | -.17 | <.001 |
| School achievement: maths | .20 | <.001 |
| School achievement: German | .28 | <.001 |
| School achievement: motivation | .25 | <.001 |

**Table S4: Correlations between matching variables and reading frequency before and after CBGPS weighting for the age 20 outcome analyses**

| **Matching variable** | **Unweighted correlation** | **Weighted correlation** |
| --- | --- | --- |
| Gender | .21 | <.001 |
| Migration status | -.16 | <.001 |
| ISEI (Occupational prestige) | .27 | <.001 |
| Age 15 internalising problems | .11 | <.001 |
| Age 15 ADHD symptoms | <.01 | <.001 |
| Age 15 general trust | .08 | <.001 |
| Age 15 self-control | -.23 | <.001 |
| Age 15 alcohol use (liquor) | -.17 | <.001 |
| Age 15 alcohol use (beer/wine) | .09 | <.001 |
| Age 15 tobacco use | -.04 | <.001 |
| Age 15 cannabis use | -.07 | <.001 |
| Age 15 reactive aggression | -.17 | <.001 |
| Age 15 physical aggression | -.23 | <.001 |
| Age 15 proactive aggression | -.11 | <.001 |
| Age 15 indirect aggression | -.14 | <.001 |
| Age 15 prosociality | .15 | <.001 |
| Age 15 delinquency | -.10 | <.001 |
| Age 15 parental involvement | .20 | <.001 |
| Age 15 social support from adults | .15 | <.001 |
| Age 15 social support from friends | <.01 | <.001 |
| Age 15 bullying victimisation | .06 | <.001 |
| Age 15 teacher bond | .08 | <.001 |
| Age 15 class bond | -.03 | <.001 |
| Age 15 school difficulties | -.13 | <.001 |
| School achievement: maths | .18 | <.001 |
| School achievement: German | .24 | <.001 |
| School achievement: motivation | .23 | <.001 |

**Figures**

**Figure S1: Key RI-CLPM parameters (standardised) for the reading and anxiety model**

**Figure S2: Key RI-CLPM parameters (standardised) for the reading and depression model**
